# Vertical blockade of the IGFR- PI3K/Akt/mTOR pathway for the treatment of hepatocellular carcinoma: the role of survivin

**DOI:** 10.1186/1476-4598-13-2

**Published:** 2014-01-03

**Authors:** Da-Liang Ou, Bin-Shyun Lee, Liang-In Lin, Jun-Yang Liou, Sheng-Chieh Liao, Chiun Hsu, Ann-Lii Cheng

**Affiliations:** 1 Graduate Institute of Oncology, College of Medicine, National Taiwan University, Taipei, Taiwan; 2 National Center of Excellence for Clinical Trial and Research, National Taiwan University Hospital, Taipei, Taiwan; 3 Department of Oncology, National Taiwan University Hospital, 7 Chung-Shan South Road, Taipei, 100, Taiwan; 4 Graduate Institute of Clinical Laboratory Sciences and Medical Biotechnology, College of Medicine, National Taiwan University, Taipei, Taiwan; 5 Institute of Cellular and System Medicine, National Health Research Institutes, Zhunan, Miaoli County, Taiwan; 6 Department of Internal Medicine, National Taiwan University Hospital, Taipei, Taiwan; 7 Graduate Institute of Toxicology, College of Medicine, National Taiwan University, Taipei, Taiwan

**Keywords:** Molecular targeted therapy, Insulin-like growth factor, NVP-AEW541, MK2206, BEZ235, RAD001

## Abstract

**Background:**

To explore whether combining inhibitors that target the insulin-like growth factor receptor (IGFR)/PI3K/Akt/mTOR signaling pathway (vertical blockade) can improve treatment efficacy for hepatocellular carcinoma (HCC).

**Methods:**

HCC cell lines (including Hep3B, Huh7, and PLC5) and HUVECs (human umbilical venous endothelial cells) were tested. The molecular targeting therapy agents tested included NVP-AEW541 (IGFR kinase inhibitor), MK2206 (Akt inhibitor), BEZ235 (PI3K/mTOR inhibitor), and RAD001 (mTOR inhibitor). Potential synergistic antitumor effects were tested by median dose-effect analysis in vitro and by xenograft HCC models. Apoptosis was analyzed by flow cytometry (sub-G1 fraction analysis) and Western blotting. The activities of pertinent signaling pathways and expression of apoptosis-related proteins were measured by Western blotting.

**Results:**

Vertical blockade induced a more sustained inhibition of PI3K/Akt/mTOR signaling activities in all the HCC cells and HUVEC tested. Synergistic apoptosis-inducing effects, however, varied among different cell lines and drug combinations and were most prominent when NVP-AEW541 was combined with MK2206. Using an apoptosis array, we identified survivin as a potential downstream mediator. Over-expression of survivin in HCC cells abolished the anti-tumor synergy between NVP-AEW541 and MK2206, whereas knockdown of survivin improved the anti-tumor effects of all drug combinations tested. In vivo by xenograft studies confirmed the anti-tumor synergy between NVP-AEW541 and MK2206 and exhibited acceptable toxicity profiles.

**Conclusions:**

Vertical blockade of the IGFR/PI3K/Akt/mTOR pathway has promising anti-tumor activity for HCC. Survivin expression may serve as a biomarker to predict treatment efficacy.

## Background

The multi-kinase inhibitor sorafenib is currently standard treatment for patients with advanced-stage hepatocellular carcinoma (HCC) [1,2]. The success of sorafenib trials encouraged development of many molecular targeted agents (MTA) that aim at specific molecular derangements in cancer cells or their microenvironment for the treatment of HCC [1]. Studies focusing on molecular classification of HCC will help not only identification of potential therapeutic targets in HCC but also patient enrichment in future clinical trials [3].

The insulin-like growth factor (IGF) signaling pathway provides an important regulatory mechanism for tumorigenesis and drug resistance in many cancers, including HCC [4,5]. Evidence supporting development of inhibitors targeting the IGF signaling pathway for HCC treatment is three folds. Firstly, many previous studies linked IGF signaling activity to HCC pathogenesis, tumor angiogenesis, and drug resistance [6-9]. Secondly, preclinical studies have shown that the efficacy of anti-cancer therapy for HCC can be improved by inhibiting the IGF signaling pathway in HCC cells [10-12]. Thirdly, clinical trials of patients with advanced HCC indicated that serum IGF levels can help predict efficacy of anti-angiogenic therapy [13]. However, although many inhibitors of IGF receptors (IGFR) have been developed and promising antitumor activity has been demonstrated in pre-clinical studies, clinical studies showed very limited efficacy of IGFR inhibitors as single-agent therapy in solid tumors, including HCC [14,15].

An important mechanism of resistance to IGFR inhibitors is the compensatory activation of related signaling pathways. The PI3K/Akt/mTOR pathway is the most extensively studied and Akt signaling has been demonstrated to be a critical mechanism of drug resistance in HCC cells [16]. Targeting a single point in these pivotal signaling pathways will induce compensatory up- or down-stream activation and result in drug resistance [17,18]. To overcome this compensatory activation, combination of agents targeting more than one molecule in a signaling pathway, known as vertical blockade, were extensively tested in multiple preclinical models and synergistic anti-cancer efficacy has been demonstrated [19-21].

In this study, we sought to clarify whether combining inhibitors that target the IGFR and the PI3K/Akt/mTOR signaling pathway could enhance therapeutic efficacy in HCC. The optimal combination of the potential synergistic antitumor activities between IGFR and PI3K/Akt/mTOR inhibitors were explored. Survivin, an apoptosis-inhibitory protein that is over-expressed in multiple cancer types and plays critical roles in regulating apoptosis, cell proliferation and survival, was found to be a direct downstream target of the PI3K/Akt/mTOR pathway. The role of survivin in mediating the anti-tumor efficacy of IGFR/PI3K/Akt/mTOR vertical blockade was also examined.

## Methods and materials

### Cell culture and reagents

The HCC cell lines tested in this study included PLC/PRF/5 (PLC5), Hep3B (from American Type Culture Collection), and Huh7 (from Health Science Research Resources Bank, Japan). Cells were cultured in Dulbecco’s modified Eagle’s medium supplemented with 10% fetal bovine serum (FBS), penicillin (100 units/mL), and streptomycin (100 μg/mL). Primary human umbilical venous endothelial cells (HUVEC), which were used to test the anti-angiogenic activity of the drug combinations, were cultured as previously described [20]. These cells were maintained in a humidified incubator under 5% CO_2_ at 37°C.

The molecular targeted agents (MTAs) tested in this study included NVP-AEW541 (IGFR inhibitor, Novartis), MK2206 (Akt inhibitor, MSD), BEZ235 (PI3K/mTOR inhibitor, Novartis), and RAD001 (mTOR inhibitor, Novartis). For in vitro experiments, the drugs were dissolved in DMSO and the final DMSO concentration was kept below 0.1%. For in vivo experiments, the following the optimal dosage were used: NVP-AEW541 (dissolved in 25 mmol/L tartaric acid, 30 mg/kg, gavage) once per day for 25 days based on previous studies [22,23], BEZ235 (dissolved in 10% NMP (1-methyl-2-pyrrolidone)/PEG300 90%, 25 mg/kg, gavage) once per day for 25 days based on previous studies [24,25], and MK2206 (dissolved in 30% captisol, 100 mg/kg, i.p.) once per week for 4 weeks based on previous studies [26,27]. The antibodies used for Western blotting, and immunohistochemical staining included anti-Myc Tag (Origene, Rockville, MD), p-Akt, caspase 3, p-GSK3β, GSK3β, p-P70S6K, P70S6K, p-4EBP-1, Mcl-1, Bim, DR-5, FADD (Cell Signaling Technology, Danvers, MA), Bcl-2, Bad, Bax, survivin, GAPDH, and actin (Santa Cruz Biotechnology, Santa Cruz, CA).

### Tests of in vitro anti-tumor efficacy

Cell viability was assessed by MTT (3-(4, 5-dimethylthiazol-2-yl)-2, 5- diphenyltetrazolium bromide) assay. The IC_50_ values after drug treatment were calculated using CompuSyn software (ComboSyn, Paramus, NJ) based on changes in absorbance as determined by spectrophotometry (DTX 880; Beckman Coulter, Fullerton, CA). Apoptotic cell death was measured by flow cytometry (sub-G1 fraction analysis) and Western blotting analysis (caspase activation, PARP cleavage). The potential synergistic antitumor effects between different drug treatments were determined by median dose-effect analysis using the combination index (CI)-isobologram method, as previously described [20].

### Identification of pertinent downstream mediators of anti-tumor efficacy

The activity of IGFR and the downstream PI3K/Akt/mTOR signaling pathways was determined by Western blotting analysis. To explore potential synergistic effects between IGFR inhibition and other MTAs on the expression of pertinent apoptosis-related proteins, we used a human apoptosis array (Proteome Profiler™, R&D Systems, Minneapolis, MN), which could simultaneously measure the relative levels of expression of 35 apoptosis-related proteins (Additional file 1: Figure S1 for the list of the apoptosis-related proteins analyzed). Signals were visualized using a UVP Imaging System (UVP, Upland, CA) or with X-ray film. Candidate molecules were selected based on their expression levels after drug treatment and by correlation with in vitro antitumor effects. The expression levels of these candidate molecules after drug treatment were confirmed by Western blotting analysis.

To explore the biological roles of the candidate molecules, small interfering RNA (siRNA) knockdown and transient over-expression were used in combination with MTAs treatment. The treated HCC cells were then collected for Western blotting or flow cytometry analysis. For the studies of survivin as the downstream mediator of IGFR/AKT/mTOR vertical blockade, si-survivin (catalog number L-003459-00-0005) and scrambled nonspecific (negative-control) siRNA (catalog number D-001810-10-20) were purchased from Thermo Scientific (Dharmacon Division). HCC cells were transfected with siRNA using siPORTNeoFx transfection reagent (Ambion, Austin, TX) and then treated with MTAs. Over-expression of survivin was done by transiently transfecting the pCMV6-Myc-DKK-BIRC5 (survivin) vector (RC205935; Origene Technologies) or empty vector (pCMV6; Origene Technologies) into HCC cells using PolyJet transfection reagent (Signagen. Laboratories, Ijamsville, MD).

### Tests of in vivo anti-tumor efficacy

Tumor xenograft experiments were performed using male BALB/c athymic (nu+/nu+) mice. Hep3B and Huh7 cells were inoculated subcutaneously at 1 × 10^6^ cells. The protocol for the xenograft experiments was approved by the Institutional Animal Care and Use Committee of the College of Medicine, National Taiwan University, and conformed to the criteria outlined in the Guide for the Care and Use of Laboratory Animals prepared by the National Academy of Sciences and published by the National Institutes of Health. The mice were randomized to different MTA treatment groups when tumor volume reached approximately 100 mm^3^ (volume [mm^3^] = [width]^2^ × length × 0.5) (animal number n ≥ 5 in each group). NVP-AEW541 and BEZ235 treatments were administered daily by gavage. MK2206 was administered weekly by intraperitoneal injection. Tumor volume and body weight were recorded every 5 days. After MTAs treatment, fresh-frozen tumor samples were collected for determination of levels of various proteins by Western blotting. After drug treatment, formalin-fixed, paraffin-embedded tumor samples were collected for immunohistochemical analysis of protein expression and tumor angiogenesis. TUNEL assay was performed to measure the extent of tumor cell apoptosis, as previously described [20].

### Statistical analysis

All data used were representative of at least three independent experiments. Quantitative data are expressed as mean ± SD. Comparisons were analyzed using the Student’s *t* test and ANOVA. Significance was defined as p < 0.05.

## Results

### In vitro anti-tumor efficacy of IGFR/PI3K/Akt/mTOR inhibition

**Figure 1:**
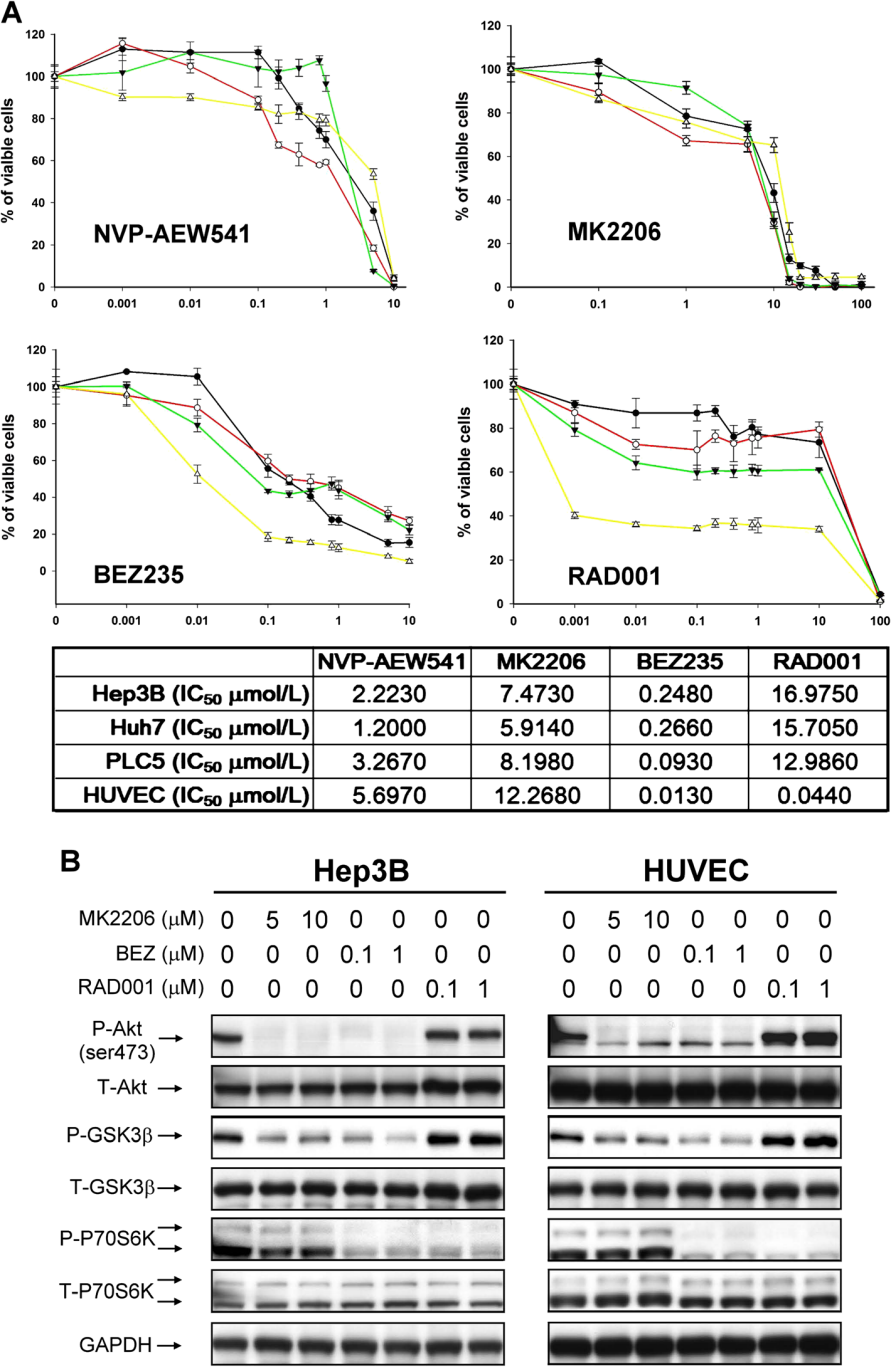
**Growth-inhibitory and downstream signaling effects of molecular targeted agents (NVP-AEW-541, IGFR inhibitor; MK2206, Akt inhibitor; BEZ235, PI3K/mTOR inhibitor; RAD001, mTOR inhibitor) on HCC cells and HUVECs. (A)** IC_50_ of HCC cell lines and HUVECs after drug treatments. Cells in 96-well plates were treated with drugs at the indicated concentrations for 72 h, and cell viability was assessed by MTT assay. Points, mean averages (n = 3); bars, SD. **(B)** Effects on Akt, GSK3β, P70S6K phosphorylation were examined by Western blotting in HCC cells and HUVECs after 24-hour drug treatments at the indicated concentrations.

The growth-inhibitory effects of NVP-AEW541 (IGFR inhibitor), MK2206 (Akt inhibitor), BEZ235 (PI3K/mTOR dual inhibitor), and RAD001 (mTOR inhibitor) on HCC cells and HUVEC were shown in Figure 1A. The response of the HCC cell lines tested to individual MTAs did not differ significantly from one another. BEZ235 appeared to be the most potent inhibitor of PI3K/Akt/mTOR signaling activity (Figure 1B). BEZ235 inhibited Akt, GSK3β, and P70S6K phosphorylation at submicromolar range, consistent with its growth-inhibitory effects. On the other hand, although RAD001 inhibited the downstream P70S6K phosphorylation at submicromolar levels, the Akt and GSK3βphosphorylation appeared increased after RAD001 treatment, suggesting compensatory activation of upstream signaling activities (Figure 1B). This finding may explain the relatively poor growth-inhibitory effects of RAD001 in the HCC cells tested (IC50 > 10 μM).

**Figure 2:**
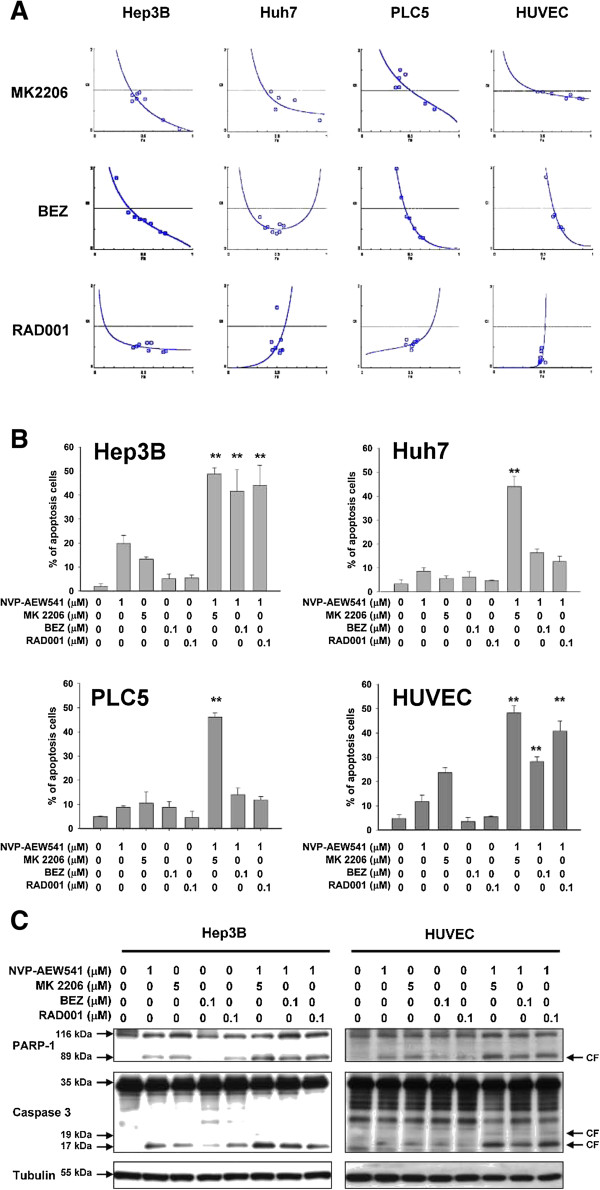
**Synergistic growth-inhibitory and apoptosis-inducing effects between the IGFR inhibitor NVP-AEW541 and PI3K/Akt/mTOR inhibitors (MK2206, BEZ235, and RAD001). (A)** Median dose-effect analysis of synergistic growth-inhibitory effects. Growth inhibition was measured by MTT assay. CI was calculated using the CI-isobologram method; CI = 1, additive effect; CI < 1, synergistic effect; CI > 1, antagonistic effect. The concentrations of the drug used for MTT assay and the original CI (combination index) values of each drug combination were summarized in Additional file 1: Table S1. **(B and C)** Synergistic apoptosis-inducing effects between NVP-AEW541 and MK2206, BEZ235, and RAD001 in HCC cells and HUVECs measured by flow cytometry (sub-G1 fraction analysis, **B)** and by PARP cleavage and caspase3 activation (Western blotting, **C)**. Columns, mean averages of three independent experiments; bars, SD. **, p < 0.01 compared with cells treated with a single inhibitor.

To investigate the potential synergistic antitumor effects of vertical blockade of the IGFR/PI3K/Akt/mTOR signaling pathway, median effect analysis was performed to measure the combination index (CI) of different treatments combining NVP-AEW541, MK2206, BEZ235, and RAD001, with CI values <1 indicating synergy (Figure 2A). Synergistic growth-inhibitory effects were seen for most of the combinations tested in all three HCC cell lines and in HUVECs. Synergistic apoptosis-inducing effects, measured by flow cytometry (sub-G1 fraction analysis) and Western blotting (PARP cleavage and caspase 3 activation), were most consistent when NVP-AEW541 was combined with the Akt inhibitor MK2206 (Figure 2B and C). BEZ235 and RAD001 could enhance apoptosis in Hep3B and HUVECs only when combined with NVP-AEW541 (Figure 2B).

### Survivin is an important downstream mediator of anti-tumor synergy

**Figure 3:**
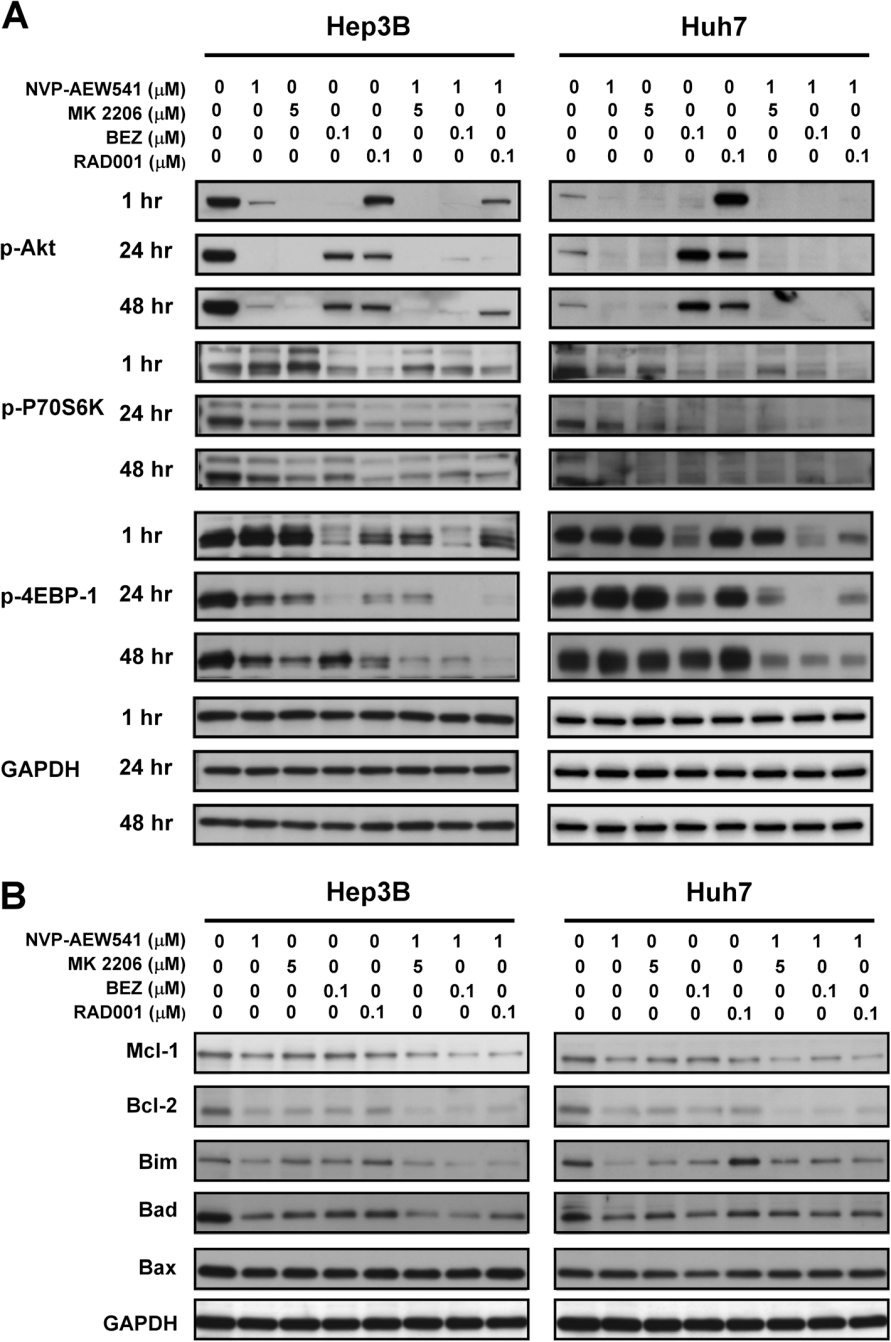
**The effects of combinations of NVP-AEW541 with MK2206, BEZ235, or RAD001 inhibitors on downstream signaling activity (A) and apoptosis-related proteins (B) in HCC cells.** Effects on phosphorylation of Akt, P70S6K, and 4EBP-1 were examined by Western blotting. HCC cells (Hep3B and Huh7) were treated with or without NVP-AEW541 and MK2206, BEZ235, or RAD001 at the indicated concentrations and times. Whole-cell lysates were subjected to Western blotting.

To explain the differential effects on apoptosis induction by different drug combination, we first compared the effects of these combinations on activity of PI3K/Akt/mTOR pathway in Hep3B and Huh7 cells. As shown in Figure 3A, all the combinations, including NVP-AEW541-MK2206, NVP-AEW541-BEZ235, and NVP-AEW541-RAD001, inhibited the phosphorylation of Akt, P70S6K, and 4EBP-1 to a similar extent in Hep3B and Huh7 cells. Therefore, the difference in apoptosis induction by different drug combination in the 2 cell lines cannot be explained by their inhibitory effects on PI3K/Akt/mTOR signaling activity alone. Similarly, the effects of these drug combinations on expression of apoptosis-related proteins, including mcl-1, bcl-2, bim, bad, and bax, were also similar in the 2 cell lines (Figure 3B).

**Figure 4:**
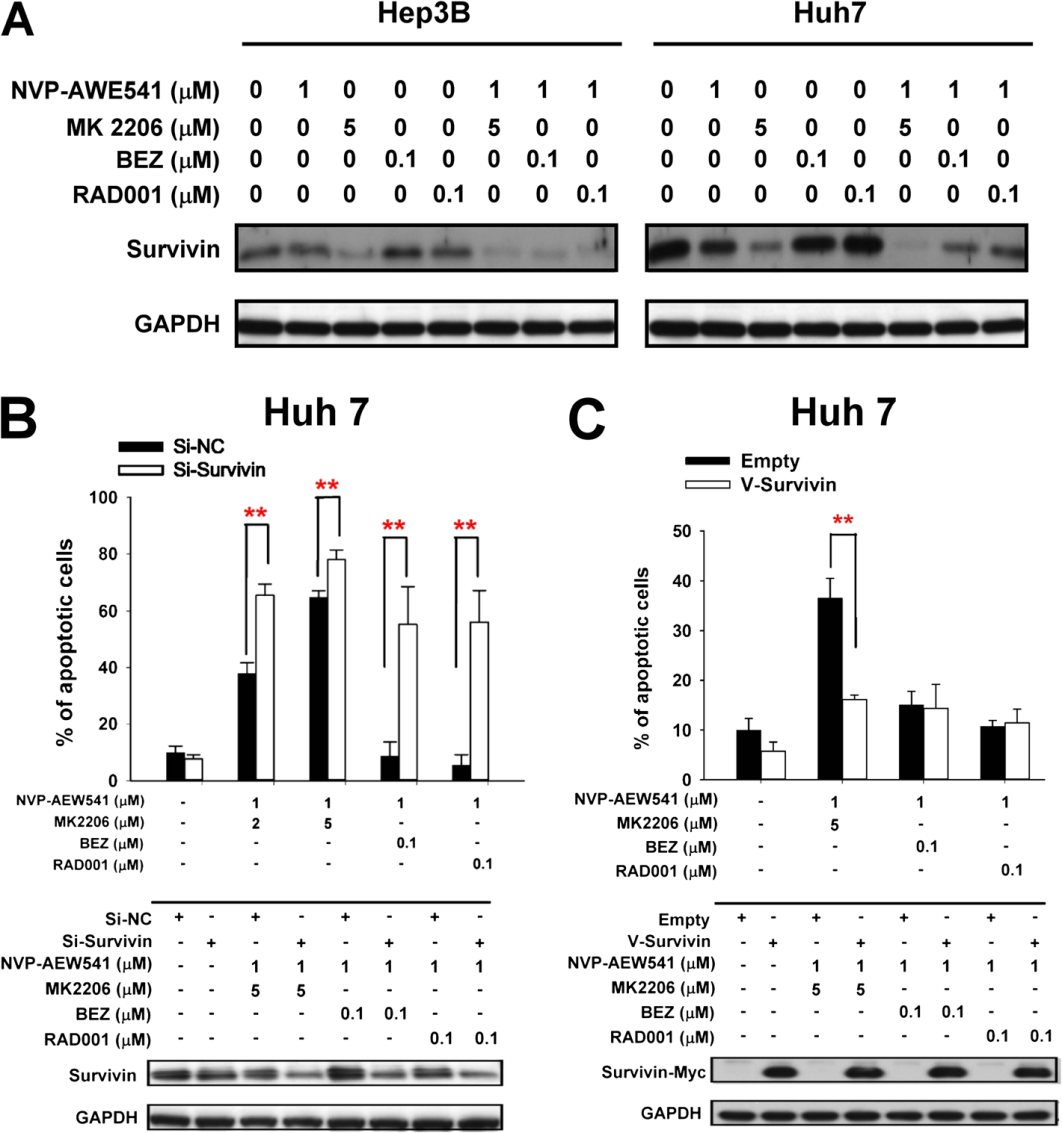
**Survivin is a possible mediator of antitumor synergy between NVP-AEW541 and MK2206, BEZ235, and RAD001. (A)** Effects between NVP-AEW541 and MK2206, BEZ235, and RAD001 on survivin expression in HCC cells. HCC cells (Hep3B and Huh7) were treated with NVP-AEW541, MK2206, BEZ235, and RAD001 at the indicated concentrations for 48 hours. Whole-cell lysates were subjected to Western blotting. **(B)** Survivin knockdown enhanced the efficacy of vertical blockade. Huh7 cells were transfected with si-survivin or scrambled siRNA for 24 hours, and then treated with different drug combination as indicated. **(C)** Huh7 cells were transfected with V-Survivin (pCMV6-Myc-DKK-survivin) or empty vectors for 8 hours, and then treated with different drug combination as indicated. Whole-cell lysates were collected for Western blotting after 48-hour drug treatment. The percentages of apoptotic cells were measured by flow cytometry after 72-hour drug treatment. Columns, mean of three independent experiments; bars, SD. **, p < 0.01.

The apoptosis protein array was then used to explore potential mediators of anti-tumor activity of different drug combinations targeting the IGFR/AKT/mTOR pathway. Survivin was identified as the candidate molecule because it showed the most consistent inhibition when NVP-AEW541 was combined with MK2206, BEZ235, or RAD001 and correlated with the anti-tumor synergy (Additional file 1: Figure S1). This finding was confirmed by Western blotting (Figure 4A). Furthermore, in Huh7 cells inhibition of survivin expression was more prominent in NVP-AEW541/MK2206 combination than in NVP-AEW541/BEZ235 and NVP-AEW541/RAD001 combinations (Figure 4A). This finding suggested that the differential effects of different drug combinations in HCC cells can be explained at least in part by their inhibitory effects on survivin expression.

The role of survivin as an important downstream mediator was further explored by siRNA knockdown and transient over-expression experiments. Suppression of survivin expression enhanced the apoptosis-inducing effects of all 3 combinations, and the effects of NVP-AEW541/BEZ235 and NVP-AEW541/RAD001 combinations were enhanced to be similar to that of NVP-AEW541/MK2206 combination (Figure 4B). In addition, over-expression of survivin decreased the apoptosis-inducing effects of NVP-AEW541/MK2206 combination, whereas the effects on NVP-AEW541/BEZ235 and NVP-AEW541/RAD001 combinations were less obvious in Huh7 (Figure 4C), and over-expression of survivin decreased the apoptosis-inducing effects of NVP-AEW541/MK2206, NVP-AEW541/BEZ235 and NVP-AEW541/RAD001 combination (Additional file 1: Figure S2). The above data supports the role of survivin as an important downstream mediator of anti-tumor synergy of vertical blockade of IGFR/PI3K/Akt/mTOR signaling pathway.

### In vivo confirmation of efficacy of vertical blockade

**Figure 5:**
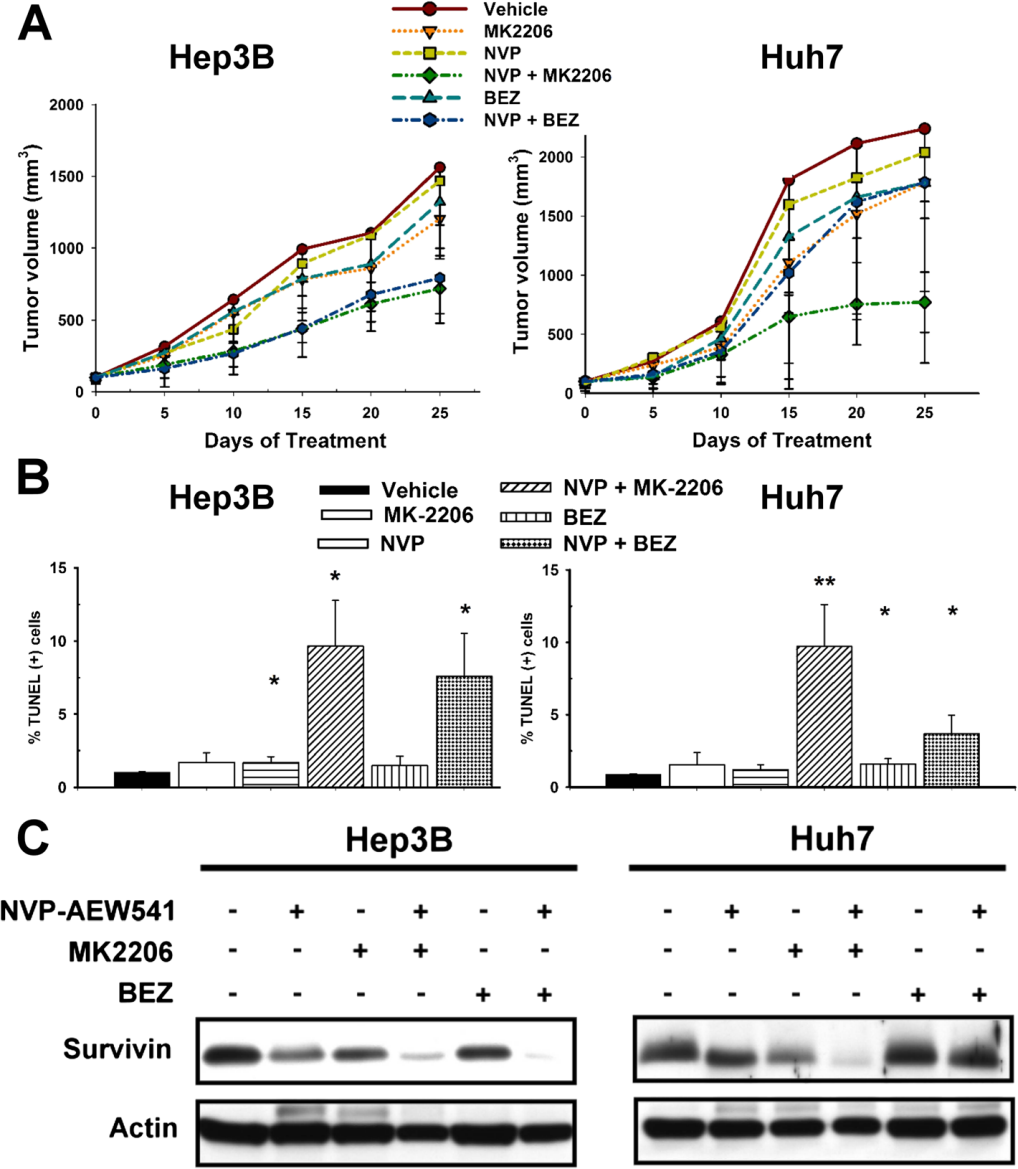
**In vivo antitumor synergy between NVP-AEW541, MK2206 and BEZ235. Hep3B and Huh7 cells were subcutaneously injected into male BALB/c athymic nude mice.** Mice were treated as indicated (NVP, NVP-AEW-541 30 mg/kg/day by gavage; MK2206, MK2206 100 mg/kg/week by intra-peritoneal injection; BEZ, BEZ235 25 mg/kg/day by gavage). Values presented are the means ± SD (n = 5 in each group). **(A)** Differences in tumor growth. **(B)** Differences in tumor cell apoptosis (TUNEL assay).*, p < 0.05; **, p < 0.01, compared with the control (vehicle-treated) group. **(C)** Difference in survivin measured by Western blotting of tumor lysates.

The antitumor synergy of vertical blockade of IGFR/PI3K/Akt/mTOR signaling pathway was also confirmed by xenograft experiments. Combination of NVP-AEW541 and MK2206 produced the most prominent anti-tumor effects in both Hep3B and Huh7 models (Figure 5A), associated with a significant increase in tumor cell apoptosis and suppression of survivin expression than single-agent treatment. By contrast, combination of NVP-AEW541 and BEZ235 produced synergistic anti-tumor effects in Hep3B but not in Huh7 models, consistent with the in vitro study results. The changes of tumor microvessel density showed similar trends in Hep3B and Huh7 models (Additional file 1: Figure S3), suggesting that the in vivo effects of vertical blockade was mainly on cancer cells per se. Drug treatment induced body weight loss, which appeared more prominent in treatments containing the Akt inhibitor MK2206 (Additional file 1: Figure S4). However, no significant abnormalities of hemogram or blood biochemistry tests were found in any of the treatment groups, suggesting that the combination treatment has an acceptable safety profile.

## Discussion

In this study we explored whether vertical blockade that targets both the IGFR and the PI3K/Akt/mTOR signaling pathway can enhance therapeutic efficacy for HCC. Our data indicated that combining the IGFR inhibitor NVP-AEW541 with inhibitors against PI3K/Akt/mTOR pathway (MK2206 or BEZ235) results in synergistic anti-tumor effects in HCC cells in vitro and in vivo. Survivin is an important mediator of this anti-tumor synergy. The combination treatments have acceptable safety profile. Therefore, further clinical trials to explore their clinical efficacy are warranted.

Vertical blockade of the PI3K/Akt/mTOR pathway has been extensively studied to overcome the compensatory activation induced by single-agent treatment [24,28]. The synergistic anti-tumor effects were associated with a more sustained inhibition of PI3K/Akt/mTOR signaling activity and down-stream regulators of cell growth, including myc, cyclin D, and retinoblastoma (RB) protein. Synergistic anti-angiogenic effects of the vertical blockade strategy have also been demonstrated in both in vitro and in vivo. It should be clarified whether the more sustained inhibition of PI3K/Akt/mTOR signaling activity is also associated with more treatment-related toxicities in cancer patients. The synergistic apoptosis-inducing effects in normal cells like HUVEC also implied potential synergistic toxicity. Early-phase clinical trials of agents targeting single points along the IGFR/PI3K/Akt/mTOR pathway reported only mild to moderate adverse events, including hyperglycemia, fatigue, skin rashes and gastrointestinal toxicities [29]. While pre-clinical studies of various combinations of these agents did not report excessive overlapping toxicity profiles, the safety of long-term use of the combination treatments in cancer patients must be carefully evaluated.

Another important issue in designing clinical trials for MTAs is patient enrichment, i.e., selection of patients who will most likely benefit from the MTAs under evaluation. Activation of IGFR and PI3K/Akt/mTOR pathways has been shown to indicate rapid tumor progression and poor prognosis in HCC [30]. Activation of this pathway is usually determined by immunohistochemical studies of protein phosphorylation in HCC tumor tissue, because genetic aberrations of IGFR and PI3K/Akt/mTOR pathways are rare in HCC [31,32]. However, such immunohistochemical studies are not reliable in HCC due to protein instability and difficulty in standardization of tissue processing [33,34]. The lack of reliable biomarkers for patient enrichment may partly explain the lack of success of IGFR inhibitors in previous clinical trials [4,14,15].

The correlation of survivin suppression and anti-tumor synergy found in our study suggest that survivin expression may be a good biomarker for treatment targeting the IGFR/PI3K/Akt/mTOR pathway. Future clinical trials may integrate evaluation of survivin in HCC tumor tissue to clarify whether baseline levels of survivin expression can serve as predictive biomarkers for treatment efficacy. Survivin has been reported as a direct downstream target of the PI3K/Akt/mTOR pathway and plays an important role in induction of drug resistance [35,36]. In this study, although vertical blockade produced sustained suppression of signaling activities in the IGFR/PI3K/Akt/mTOR pathway of all the HCC cells tested, the impact on survivin expression and subsequent anti-tumor synergy varied among different combinations and cell lines. It has been demonstrated that vertical blockade of the PI3K/Akt/mTOR pathway can induce profound changes in gene expression independent of PI3K/Akt/mTOR signaling activities [28]. Moreover, survivin expression is regulated by a complex intra-cellular signaling network involving the PI3K/Akt/mTOR, RAF/MAPK/ERK, NFκB, and hypoxia-inducible factor (HIF)-1α [37]. Therefore, it is likely that the effects of IGFR/PI3K/Akt/mTOR vertical blockade on survivin expression and anti-cancer efficacy will be affected by the interactions of the cellular signaling network. Survivin expression, which acts as an integration of the cellular signals determining cellular proliferation, survival, and drug resistance, will therefore become a more direct biomarker to predict and monitor treatment response.

## Conclusion

In summary, our data indicates that combination of inhibitors targeting IGFR and the PI3K/Akt/mTOR pathway can improve treatment efficacy for HCC in vitro and in a xenograft mouse model in vivo. Survivin expression can serve as a biomarker to predict the anti-tumor synergy. The efficacy and safety of the combination treatments should be explored by future clinical trials.

## Abbreviations

IGFR: Insulin-like growth factor receptor; PI3K: Phosphatidylinositol 3-kinase; Akt: Protein kinase B; mTOR: Mammalian target of rapamycin; HUVECs: Human umbilical venous endothelial cells; MTA: Molecular targeted agents; DMSO: Dimethyl sulfoxide; PBS: Phosphate buffered saline; HCC: Hepatocellular carcinoma; CI: Combination index

## Competing interests

Dr Ann-Lii Cheng is a consultant for Sanofi-Aventis Inc; Pfizer; Bayer Schering Pharma; Bristol-Myers Squibb (Taiwan) Ltd; Boehringer Ingelheim Taiwan Limited; and Novartis Inc. Dr Chiun Hsu received a research grant from Celgene. Other authors have no relevant financial interests related to this article.

## Authors’ contributions

DL participated in study design, performing experiments, data analysis and drafting of manuscript. BS and SC performed the experiments. LI and JY participated in study design and discussion. CH wrote the final version of the manuscript. CH and AL organized and supervised the whole study. All authors read and approved the final manuscript.

## Supplementary Material

Additional file 1: Figure S1 (A) The list of apoptosis-related proteins measured by the Human Apoptosis Array kit (Proteome ProfilerTM, R&D Systems, Minneapolis, MN). (B) Evaluation of effects of different drug combinations on expression of apoptosis-related proteins. **Figure S2.** Survivin is a possible mediator of antitumor synergy between NVP-AEW541 and MK2206, BEZ235, and RAD001. **Figure S3.** In vivo tumor angiogenesis between NVP-AEW541, and MK2206 and BEZ235 inhibitors. **Figure S4.** Assessment of the in vivo toxicities. **Table S1.** Median dose effect analysis of synergistic growth inhibitory effects of NVP-AEW541/ MK2206, NVP-AEW541/ BEZ235 and NVP-AEW541/ RAD001. Click here for file
