# Supplementary material for: Vertical blockade of the IGFR- PI3K/Akt/mTOR pathway for the treatment of hepatocellular carcinoma: the role of survivin
Source: Mol Cancer. 2014 Jan 3;13:2. doi: 10.1186/1476-4598-13-2 (PMC3882101; doi:10.1186/1476-4598-13-2)
Supplement: Additional file 1: Figure S1 — (A) The list of apoptosis-related proteins measured by the Human Apoptosis Array kit (Proteome ProfilerTM, R&D Systems, Minneapolis, MN). (B) Evaluation of effects of different drug combinations on expression of apoptosis-related proteins. Figure S2. Survivin is a possible mediator of antitumor synergy between NVP-AEW541 and MK2206, BEZ235, and RAD001. Figure S3. In vivo tumor angiogenesis between NVP-AEW541, and MK2206 and BEZ235 inhibitors. Figure S4. Assessment of the in vivo toxicities. Table S1. Median dose effect analysis of synergistic growth inhibitory effects of NVP-AEW541/ MK2206, NVP-AEW541/ BEZ235 and NVP-AEW541/ RAD001. [file 1476-4598-13-2-S1.docx]

Supplementary figure 1.

1. The list of apoptosis-related proteins measured by the Human Apoptosis Array kit (Proteome Profiler^TM^, R&D Systems, Minneapolis, MN).
2. Evaluation of effects of different drug combinations on expression of apoptosis-related proteins. Hep3B cells were treated with different drug combinations (NVP-AEW541 (1μM) + MK2206 (5μM), NVP-AEW541 (1μM) + BEZ235 (0.1μM), and NVP-AEW541 (1μM) + RAD001 (0.1μM)) for 48 hours. Whole-cell lysates were incubated with the protein array membranes according to the manufacturer’s instructions.


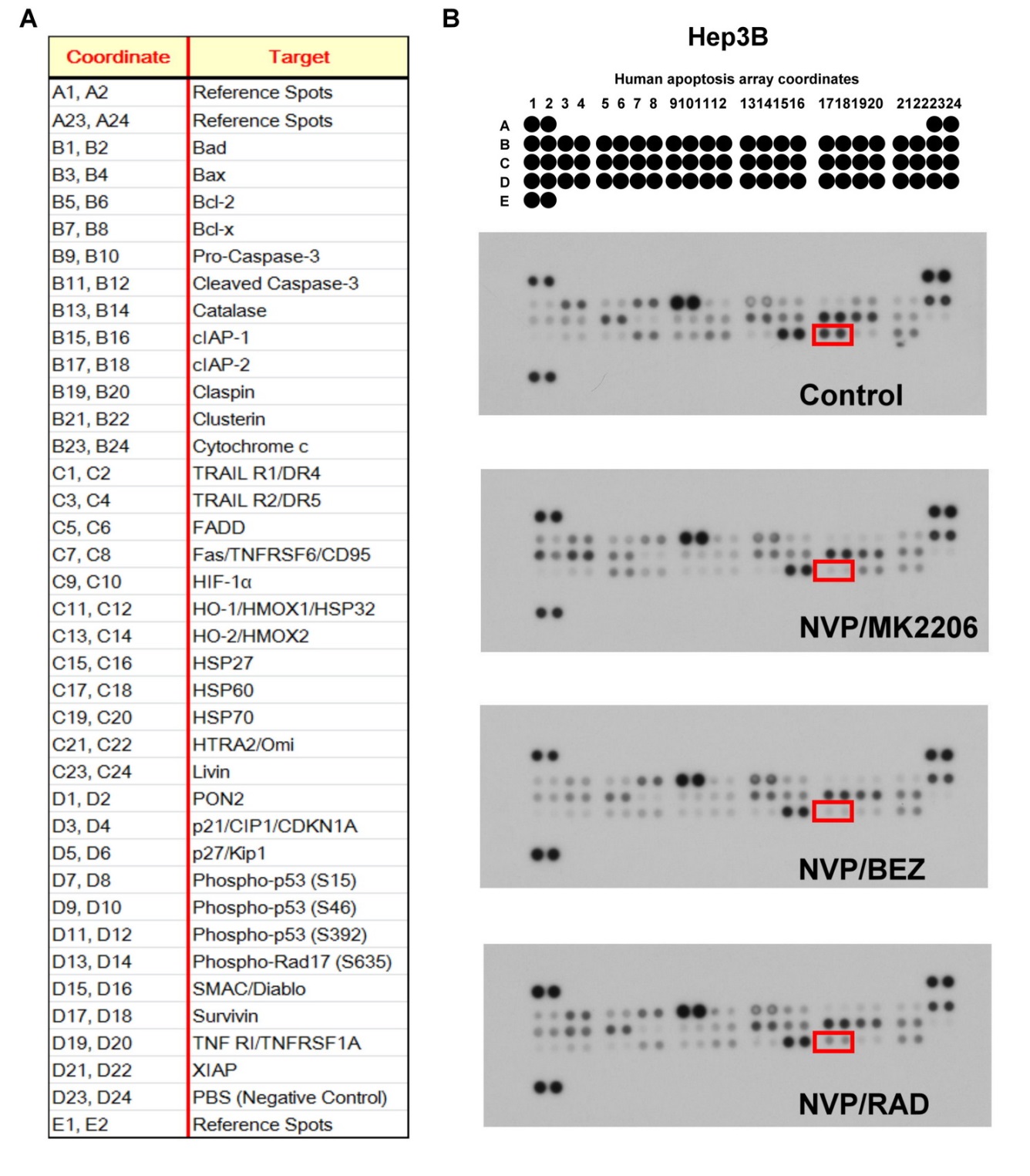


Supplementary figure 2.

Survivin is a possible mediator of antitumor synergy between NVP-AEW541 and MK2206, BEZ235, and RAD001.

Hep3B cells were transfected with V-Survivin (pCMV6-Myc-DKK-survivin) or empty vectors for 8 hours, and then treated with different drug combination as indicated. The percentages of apoptotic cells were measured by flow cytometry after 48-hour drug treatment. **, p < 0.01.


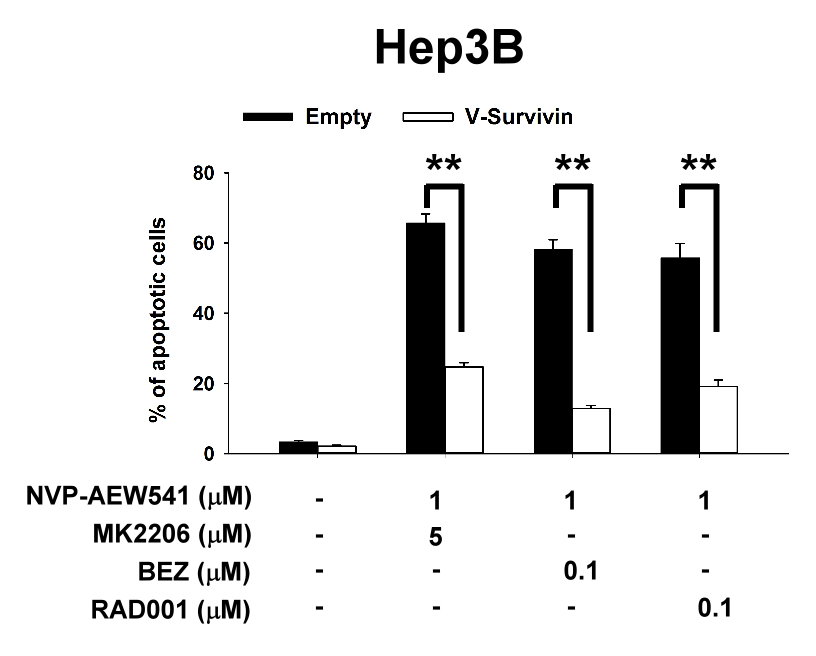


Supplementary figure 3.

In vivo tumor angiogenesis between NVP-AEW541, and MK2206 and BEZ235 inhibitors. Hep3B and Huh7 cells were subcutaneously injected into male BALB/c athymic nude mice. Mice were treated as indicated (NVP, NVP-AEW-541 30 mg/kg/day by gavage; MK2206, MK2206 100 mg/kg/week by intraperitoneal (i.p.) injection; BEZ, BEZ235 25 mg/kg/day by gavage). Tumor angiogenesis (microvessel density (MVD)).*, p < 0.05; **, p < 0.01, compared with the control (vehicle-treated) group. Values presented are the means ± SD (n = 4 in each group).


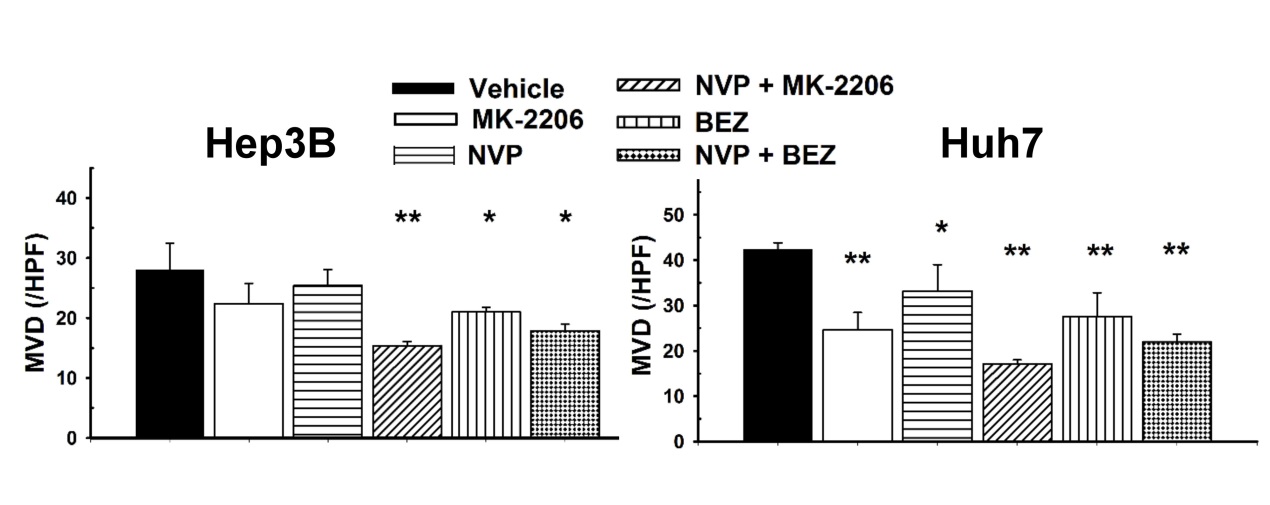


Supplementary figure 4.

Assessment of the in vivo toxicities. Male BALB/c athymic nude mice were treated as indicated (NVP, NVP-AEW-541 30 mg/kg/day by gavage; MK2206, MK2206 100 mg/kg/week by intraperitoneal (i.p.) injection; BEZ, BEZ235 25 mg/kg/day by gavage) after subcutaneous tumor implantation with Huh-7 cells. Values presented are the means ± SD (n = 5 in each group).

1. Body weight was recorded every five days.
2.
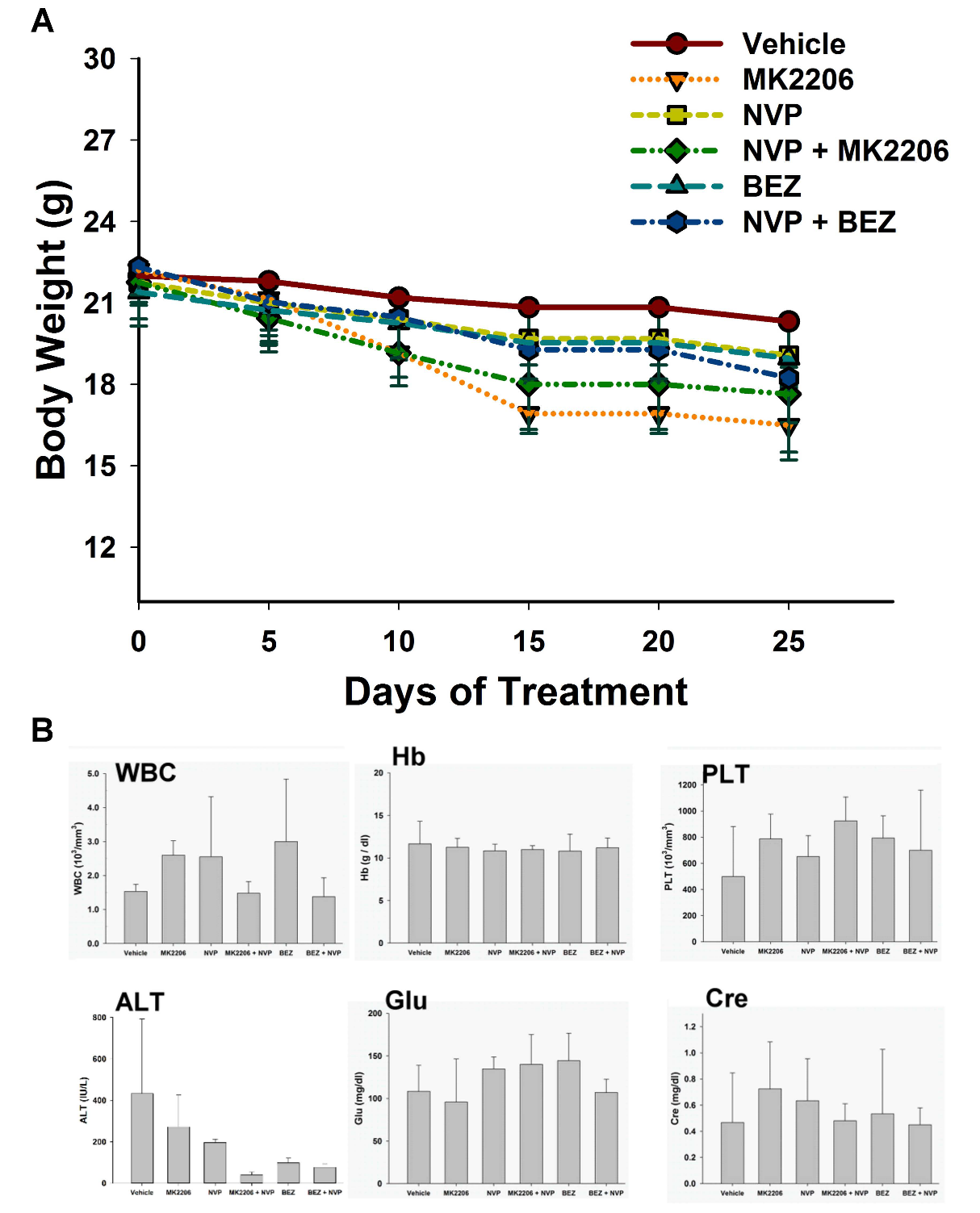
Blood samples were obtained via cardiac puncture after 28 days of drug treatment. White blood cell counts (WBC), hemoglobin (Hb), and platelet (PLT) counts were measured by a quantitative automated hematology analyzer (the Sysmex XT2000i, Sysmex America). Creatinine (Cre), alanine aminotransferase (ALT), and glucose (Glu) were measured by a quantitative automated Chemistry Analyzer (the Advia 1800, Siemens America).

B


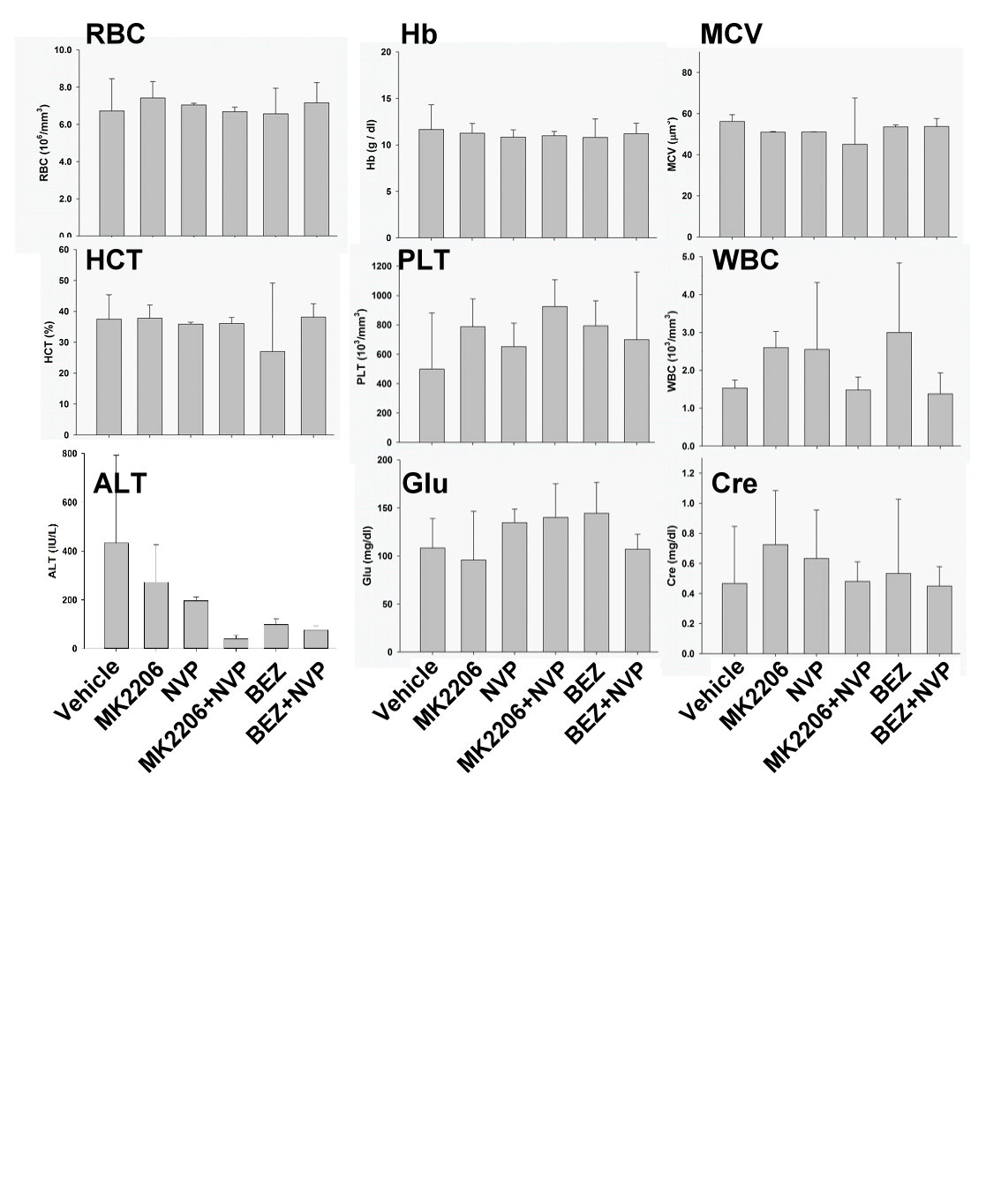


Supplementary table 1.

Median dose effect analysis of synergistic growth inhibitory effects of NVP-AEW541/ MK2206, NVP-AEW541/ BEZ235 and NVP-AEW541/ RAD001. The dose levels of different treatments combining NVP-AEW541, MK2206, BEZ235, and RAD001 used and the corresponding CI (combination index) were listed in the tables.
